# Trajectories of behavior, attention, social and emotional problems from childhood to early adulthood following extremely preterm birth: a prospective cohort study

**DOI:** 10.1007/s00787-018-1219-8

**Published:** 2018-09-07

**Authors:** Louise Linsell, Samantha Johnson, Dieter Wolke, Joan Morris, Jennifer J. Kurinczuk, Neil Marlow

**Affiliations:** 1 National Perinatal Epidemiology Unit (NPEU), Nuffield Department of Population Health, University of Oxford, Old Road Campus, Headington, Oxford, OX3 7LF UK; 2 Department of Health Sciences, Centre for Medicine, University of Leicester, University Road, Leicester, LE1 7RH UK; 3 Division of Health Sciences, Department of Psychology, Warwick Medical School, University of Warwick, Coventry, CV4 7AL UK; 4 Centre for Environmental and Preventive Medicine, Barts and The London School of Medicine and Dentistry, Queen Mary University of London, Charterhouse Square, London, EC1M 6BQ UK; 5 Medical School Building, Institute of Women’s Health, University College London, 74 Huntley Street, London, WC1E 6AU UK

**Keywords:** Extremely premature, Child behavior, Adolescent behavior, Cohort analysis, Follow-up studies

## Abstract

To investigate trajectories of behavior, attention, social and emotional problems to early adulthood in extremely preterm survivors compared to a term-born comparison group. Longitudinal analysis of a prospective, population-based cohort of 315 surviving infants born < 26 completed weeks of gestation recruited at birth in 1995, from the UK/Republic of Ireland, and a term-born comparison group recruited at age 6. The parent-report Strengths and Difficulties Questionnaire was completed at age 6, 11, 16 and 19 years. The Total Behavioral Difficulties Score was 4.81 points higher in extremely preterm individuals compared to their term-born peers over the period (95% CI 3.76–5.87, *p* < 0.001) and trajectories were stable in both groups. The impact of difficulties on home life, friendships, school or work and/or leisure activities was greater in the EPT group (RR 4.28, 95% CI 2.89–6.35, *p* < 0.001), and hyperactivity/inattention and peer problems accounted for the largest differences. A clinically significant behavioral screen at age 2.5 was associated with a higher Total Behavioral Difficulties Score from 6 years onwards in extremely preterm participants (Mean difference 6.90, 95% CI 5.01–8.70, *p* < 0.0.01), as was moderate/severe cognitive impairment at last assessment (Mean difference: 4.27, 95% CI 2.76–5.77, *p* < 0.001). Attention, social and emotional problems in extremely preterm individuals persist into early adulthood with significant impact on daily life. A positive behavioral screen in infancy and moderate/severe cognitive impairment are associated with early adult outcomes.

**Electronic supplementary material:**

The online version of this article (10.1007/s00787-018-1219-8) contains supplementary material, which is available to authorized users.

## Introduction

Improved obstetric and neonatal care over recent decades has led to a steady increase in the survival of children born extremely preterm (EPT; < 28 weeks’ of gestation) [1], which has subsequently lead to an increased prevalence of long-term sequelae such as neurodevelopmental impairments and psychiatric disorders. Compared with term-born children, a higher prevalence of parent- and/or teacher-reported behavioral problems, in particular emotional symptoms, inattention and peer relationship problems, are well documented in school-aged children born extremely preterm [2–4]. Studies using diagnostic evaluation have also reported a significant excess of psychiatric disorders, in particular attention deficit/hyperactivity disorders (ADHD), autism spectrum disorders and emotional disorders in school-aged children born extremely and very preterm (VPT; < 32 weeks’ of gestation) [5–7]. However, recent systematic reviews of the social development of children born very preterm highlighted the paucity of research in adolescence and early adulthood, a critical period of physical, emotional and social change, and a notable lack of longitudinal data [8, 9].

Generally, it has been shown that disorders differ in their age at onset and persistence throughout childhood and adolescence into adulthood, in populations not born preterm [10, 11]. The few longitudinal studies that have investigated the prevalence of behavioral symptoms or disorders in children and adolescents who were born preterm or low birth weight have shown that the increased prevalence of problems persists over time and may have greater stability in individuals born preterm compared with those born at term or with normal birthweight [2, 12–16]. Many of these previous studies employed cross-sectional analysis techniques which are not able to detect variation in individual trajectories; or they focussed on specific disorders such as ADHD. Little is known about how symptoms in various areas of mental health change over time following preterm birth, taking into account differences in the natural onset and course of different behavioral disorders. In particular, the trajectories of behavior, attention, social and emotional problems into adulthood for extremely preterm survivors have not been studied.

We conducted a longitudinal analysis of the change in behavior, attention, social and emotional problems in EPT survivors from childhood to early adulthood in the EPICure study; the largest prospective, population-based cohort of individuals born EPT [17, 18]. The main objective of this study was to investigate trajectories of behavioral problems in EPT survivors from 6 to 19 years of age compared to those of a term-born comparison group; for behavioral problems overall, and separately for emotional, conduct, hyperactivity/inattention and peer relationship problems. Our secondary objectives were to examine the impact of a positive screen for behavioral problems in early infancy and the effect of moderate/severe cognitive impairment on trajectories of behavioral problems among individuals born EPT. Both of these factors have consistently been shown to be associated with an increased risk of poorer developmental outcomes within preterm populations [15, 19, 20].

## Method

### Participants

Recruitment and follow-up to age 11 in the EPICure cohort study has been reported in full previously [21, 22]. All infants born at 25 completed weeks of gestation or less in all 276 maternity units in the United Kingdom and the Republic of Ireland from 1st March to 31st December 1995 were identified. The 315 surviving infants at hospital discharge were invited for assessments at 2.5, 6, 11 and 19 years of age, with a brief questionnaire-based assessment at age 16. The flow of participants is displayed in Online Resource Figure S1. At 6 years, for the 204/241 (85%) children attending mainstream school, a term-born classroom control was identified, matched on age, sex and race. Of the 160 controls assessed at 6 years, 110 (69%) were reassessed at 11 years of age, and 43 replacement controls were identified if the EPT child had moved school or the original control declined further participation.

### Data collection and outcome assessment

#### Behavioral assessments

At the 2.5-year assessment, the Child Behavior Checklist (CBCL) [23] was completed by parents or carers of EPT infants. This is a 100-item behavioral screening questionnaire in which parents are requested to circle 0 if an item is not true for their child, 1 if it is somewhat or sometimes true and 2 if it is often/very true. The CBCL for 2- to 3-year olds includes six syndrome scales that are combined to give an overall Total Problem score: Anxious/depressed, Withdrawn, Aggressive, Destructive, Sleep problems and Somatic behavior. The Total Problem score can be prorated if the number of missing items is less than 10%. Scores above the 90th centile are defined as clinically significant.

At the 6-, 11-, 16- and 19-year assessments, parents or carers were asked to complete the Strengths and Difficulties Questionnaire (SDQ) [24]. The SDQ can be used as both a behavioral screening tool and dimensional measure and has excellent psychometric properties for identifying children with behavioral and emotional difficulties in clinical and community populations [25, 26]. The questionnaire comprises 25 items which are scored 0 if an item is not true, 1 if it is somewhat true and 2 if it is certainly true. The 25 items in the SDQ comprise five scales of five items each; Emotional Symptoms, Conduct Problems, Hyperactivity/Inattention, Peer Problems and Prosocial Behavior. A Total Difficulties Score is calculated by combining the first four scales (excluding the Prosocial Scale) to give a total score of 0–40, with scores of 17 and above indicating significant difficulties. Scale scores may be prorated if at least three of the five items are completed.

The SDQ also contains five supplementary items to assess the impact of any problems on the child’s home and school life, friendships and daily activities, including overall distress and social impairment. Reponses on these items are summed to generate an impact score that ranges from 0 to 10 for the parent-completed version. An overall impact score of 2–10 is classified as significant impact.

#### Developmental assessments

Development at age 2.5 years corrected for gestational age was assessed using the Bayley Scales of Infant Development—second edition [27] which produces age-standardized index scores for cognitive development and motor development. At 6- and 11-year chronological age, the Kaufman Assessment Battery for Children [28] was used, which yields an age-standardized mental processing composite score for global cognitive ability. At the 19-year assessment, the Wechsler Abbreviated Scale of Intelligence—second edition [29] was administered, generating a Full Scale IQ score. Moderate/severe cognitive impairment was defined using scores more than 2 standard deviations below the mean of the term-born control group at the last clinical assessment.

### Statistical analysis

EPT participants and term-born controls were classified according to their pattern of missing assessments: completers (no missing assessments) and non-completers (one or more missing assessments). Maternal and infant characteristics were compared between the completers and non-completers within the EPT and control group. Two-sided *p* values were calculated using Fisher’s Exact Test for binary variables and the *t* test for continuous variables.

Group mean differences in the SDQ Total Difficulties Score and subscale scores between EPT children and controls were calculated for each time point with 95% confidence intervals. Hierarchical mixed modeling was used to examine trajectories in scores from age 6 to 19 years using Stata/SE version 13.1 for Windows, treating the data as having a hierarchical structure with observations at each time point nested within each individual. This is a sensitive method for assessing change as it can test for different patterns of development (intercept, slope and curvature) and can also incorporate individuals with incomplete data. Age was fitted as a random effect and centered at 6 years to make the intercept coefficient more meaningful. A group term was added as a fixed covariate, to test for a difference in intercept between the EPT and control group. An interaction term between age and group was then added to test whether the EPT and control group varied on slope, and then a quadratic function of age to test for curvature in the trajectories. For a parameter to be retained in the model, it was required to have a *p* value < 0.05.

The risk ratios for dichotomous SDQ scores (significant difficulties versus not) and the impact score, in EPT participants compared to term-born controls, were calculated using a random-effect Poisson regression model. To investigate whether a positive behavioral screen in early infancy or moderate/severe cognitive impairment was associated with behavioral problems in childhood and adolescence, a model was fitted for the Total Difficulties Score within the EPT group only. The CBCL classification (clinical versus not clinical) at age 2.5 years and cognitive impairment at last assessment (moderate/severe versus none/mild) were added separately as fixed covariates, and then together in the same model.

Analyses were first conducted in all participants with data available at any time point, and then restricted to completers only.

## Results

### Participants

Baseline characteristics of EPT participants and term-born controls by completeness of data are shown in Online Resources Tables S1 and S2. EPT completers were more likely to have mothers who were of white ethnicity, better educated and have fathers with a non-manual occupation. They also had higher BSID-II MDI and PDI scores at 2.5 years and were less likely to be diagnosed with moderate/severe cerebral palsy than non-completers. There was no evidence of a difference in behavioral problems at 2.5 years between completers and non-completers as assessed by parent-reported CBCL scores. There were no statistically significant differences between completers and non-completers in the control group.

### SDQ total difficulties score in extremely preterm individuals and term-born controls

**Fig. 1:**
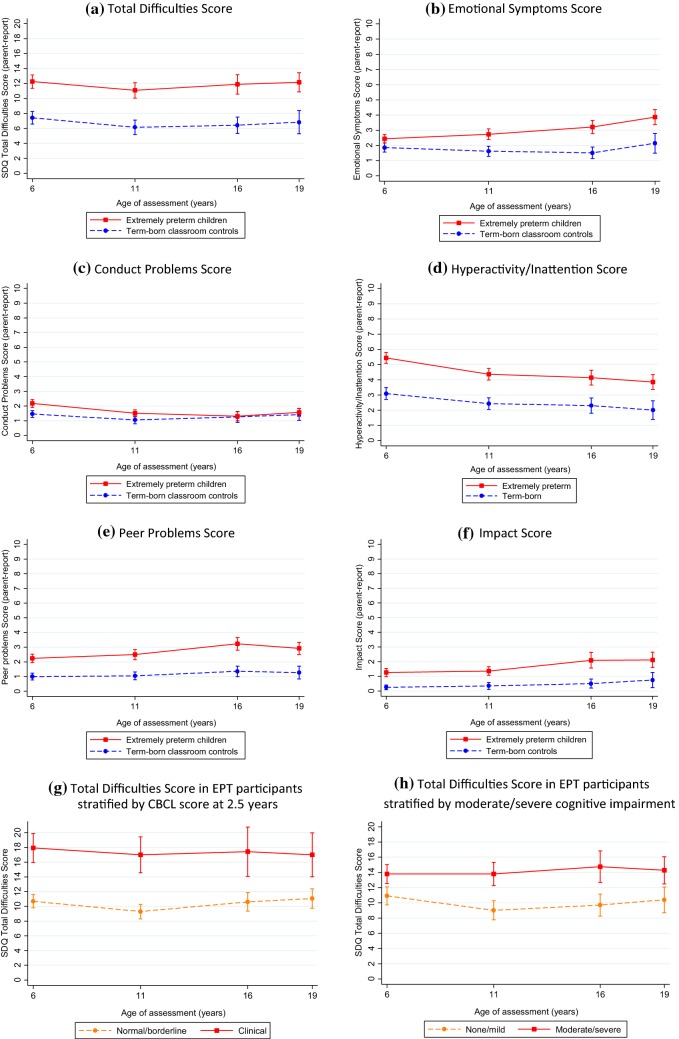
Mean plus 95% confidence intervals for SDQ Total Difficulties and subscale scores in extremely preterm participants and term-born controls at age 6, 11, 16 and 19

**Fig. 2:**
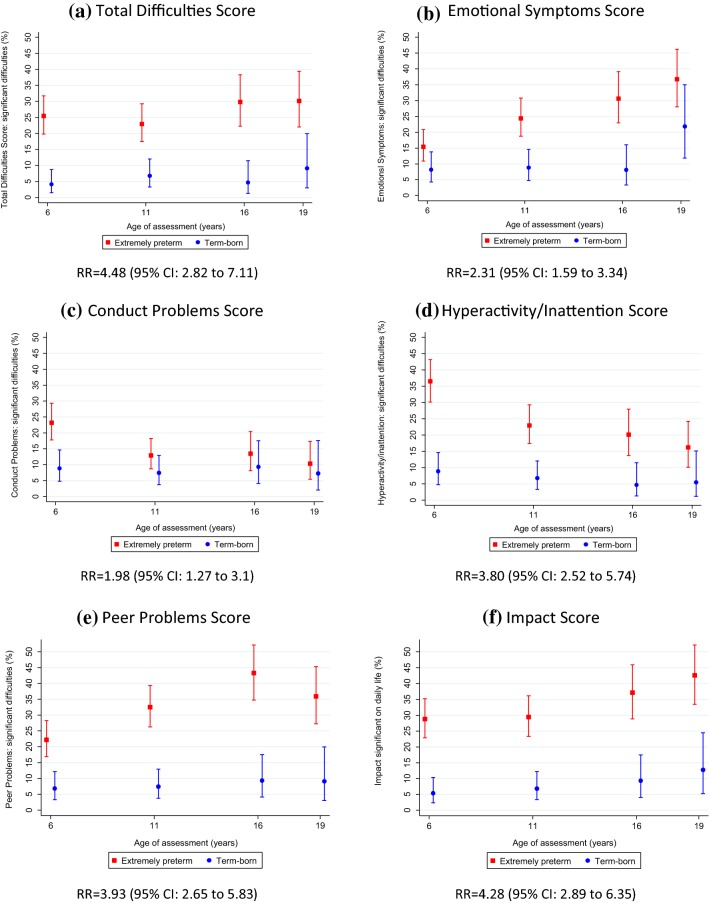
Percentage in abnormal range and 95% confidence intervals for SDQ Total Difficulties and subscale scores in the extremely preterm participants and term-born controls at age 6, 11, 16 and 19

The mean Total Difficulties Score with 95% confidence interval at each age are presented in Table 1 and displayed in Fig. 1a. The estimated coefficients and 95% confidence intervals are presented in Table 2. The predicted Total Difficulties Scores of EPT participants were 4.81 points above their term-born peers at age 6 (95% CI 3.76–5.87, *p* < 0.001) (Table 2); almost one standard deviation above the control group (SD 5.1), and trajectories (longitudinal course) were similar in both groups. The proportion of participants with symptoms classified as clinically significant for the total score and each subscale are presented in Fig. 2a. EPT individuals were at increased risk of having overall difficulties in the clinically significant range compared to their term-born peers (Risk ratio 4.48, 95% CI 2.82–7.11).

**Table 1:** Mean difference plus 95% confidence intervals for parent-report SDQ Total Difficulties and subscale scores in extremely preterm participants and term-born controls by age of assessment

	Age 6 years^a^		Age 11 years		Age 16 years		Age 19 years^b^	
	EPT (*n* = 222)	Control (*n* = 148)	EPT (*n* = 209)	Control (*n* = 148)	EPT (*n* = 134)	Control (*n* = 86)	EPT (*n* = 117)	Control (*n* = 55)
Age at assessment								
Mean [SD]	6.3 [0.5]	6.1 [0.5]	10.9 [0.4]	11.0 [0.6]	17.1 [0.3]	17.0 [0.3]	19.3 [0.5]	19.2 [0.6]
Total difficulties score								
Mean (95% confidence interval)	12.3 (11.4–13.1)	7.4 (6.6–8.3)	11.1 (10.1–12.1)	6.2 (5.2–7.1)	11.9 (10.6–13.2)	6.4 (5.3–7.5)	12.2 (10.9–13.5)	6.8 (5.3–8.4)
Mean difference (95% confidence interval)	4.8 (3.6–6.1)		4.9 (3.5–6.4)		5.5 (3.6–7.3)		5.3 (3.2–7.5)	
Abnormal range (17–40), *n* (%)	56 (25.5)	6 (4.1)	48 (23.0)	10 (6.8)	40 (29.9)	4 (4.7)	35 (30.2)	5 (9.1)
Emotional symptoms								
Mean (95% confidence interval)	2.4 (2.2–2.7)	1.9 (1.6–2.2)	2.7 (2.4–3.1)	1.6 (1.3–2.0)	3.2 (2.8–3.6)	1.5 (1.1–1.9)	3.9 (3.4–4.4)	2.1 (1.5–2.8)
Mean difference (95% confidence interval)	0.6 (0.2–1.0)		1.1 (0.6–1.6)		1.7 (1.1–2.3)		1.7 (0.9–2.6)	
Abnormal range (5–10), *n* (%)	34 (15.4)	12 (8.2)	51 (24.4)	13 (8.8)	41 (30.6)	7 (8.1)	43 (36.8)	12 (21.8)
Conduct problems								
Mean (95% confidence interval)	2.2 (1.9–2.4)	1.5 (1.2–1.7)	1.5 (1.3–1.7)	1.1 (0.8–1.3)	1.3 (1.0–1.6)	1.3 (0.9–1.6)	1.6 (1.3–1.8)	1.4 (1.0–1.8)
Mean difference (95% confidence interval)	0.7 (0.3–1.1)		0.5 (0.1–0.8)		0.1 (− 0.4 to 0.5)		0.2 (− 0.3 to 0.6)	
Abnormal range (4–10), *n* (%)	51 (23.2)	13 (8.8)	27 (12.9)	11 (7.4)	18 (13.4)	8 (9.3)	12 (10.3)	4 (7.3)
Hyperactivity/inattention								
Mean (95% confidence interval)	5.4 (5.1–5.8)	3.1 (2.7–3.5)	4.4 (4.0–4.7)	2.4 (2.1–2.8)	4.1 (3.6–4.6)	2.3 (1.8–2.8)	3.9 (3.4–4.3)	2.0 (1.4–2.6)
Mean difference (95% confidence interval)	2.3 (1.8–2.9)		1.9 (1.4–2.5)		1.8 (1.1–2.6)		1.8 (1.0–2.7)	
Abnormal range (7–10), *n* (%)	81 (36.5)	13 (8.8)	48 (23.0)	10 (6.8)	27 (20.2)	4 (4.7)	19 (16.2)	3 (5.5)
Peer problems								
Mean (95% confidence interval)	2.2 (1.9–2.5)	1.0 (0.8–1.2)	2.5 (2.1–2.8)	1.0 (0.8–1.3)	3.2 (2.8–3.7)	1.4 (1.0–1.7)	2.9 (2.5–3.3)	1.3 (0.8–1.7)
Mean difference (95% confidence interval)	1.2 (0.8–1.6)		1.4 (1.0–1.9)		1.9 (1.3–2.5)		1.6 (1.0–2.3)	
Abnormal range (4–10), *n* (%)	49 (22.2)	10 (6.8)	68 (32.5)	11 (7.4)	58 (43.3)	8 (9.3)	42 (35.9)	5 (9.1)
Prosocial behavior								
Mean (95% confidence interval)	7.5 (7.2–7.8)	8.4 (8.1–8.6)	8.3 (8.0–8.6)	9.0 (8.8–9.2)	7.8 (7.4–8.2)	8.4 (8.0–8.8)	7.6 (7.1–8.1)	8.3 (7.8–8.8)
Mean difference (95% confidence interval)	− 0.9 (− 1.3 to − 0.4)		− 0.8 (− 1.2 to − 0.3)		− 0.6 (− 1.2 to − 0.02)		− 0.7 (− 1.5 to 0.07)	
Abnormal range (0–4), *n* (%)	18 (8.3)	0 (0.0)	16 (7.7)	0 (0.0)	17 (12.7)	4 (4.7)	17 (14.7)	3 (5.6)
Impact score^c^								
Mean (95% confidence interval)	1.3 (1.0–1.5)	0.3 (0.1–0.4)	1.4 (1.1–1.7)	0.3 (0.1–0.6)	2.1 (1.6–2.6)	0.5 (0.2–0.8)	2.1 (1.6–2.6)	0.8 (0.2–1.3)
Mean difference (95% confidence interval)	1.0 (0.6–1.4)		1.0 (0.6–1.4)		1.6 (0.9–2.3)		1.4 (0.6–2.2)	
Abnormal range (2–10), *n* (%)	63 (28.8)	8 (5.4)	61 (29.5)	10 (6.9)	49 (37.2)	8 (9.3)	49 (42.6)	7 (12.7)

Higher scores indicate more behavioral problems (except for Prosocial Behavior where a higher score indicates greater sociability)

^a^4 EPT participants and 2 controls had at least one missing subscale score at age 6 years

^b^1 EPT child and 1 control had at least one missing subscale score at age 19 years

^c^Missing data at 6 years: 3 EPT, 11 years: 3 EPT and 2 control, 16 years: 8 EPT, 19 years: 2 EPT

**Table 2:** Mixed model analysis of the SDQ total difficulties score and subscales

Extremely preterm participants compared to term-born controls										
Parameter	SDQ total difficulties score (*n* = 443)		Emotional symptoms score (*n* = 443)		Conduct problems score (*n* = 443)		Hyperactivity/inattention score (*n* = 444)		Peer problems score (*n* = 443)	
	Estimate	95% CI	Estimate	95% CI	Estimate	95% CI	Estimate	95% CI	Estimate	95% CI
Constant	7.62	(6.87 to 8.38)	1.85	(1.58 to 2.13)	1.50	(1.29 to 1.72)	3.13	(2.75 to 3.50)	0.95	0.75 to 1.15)
EPT	4.81	(3.76 to 5.87)	0.67	(0.29 to 1.05)	0.75	(0.42 to 1.07)	2.33	(1.84 to 2.83)	1.37	(1.05 to 1.68)
AGE	− 0.40	(− 0.57 to − 0.23)	− 0.08	(− 0.15 to − 0.01)	− 0.14	(− 0.20 to − 0.09)	− 0.18	(− 0.26 to − 0.11)	0.05	(0.03 to 0.07)
EPT*AGE	–	–	0.06	(0.01 to 0.11)	− 0.05	(− 0.09 to − 0.02)	− 0.07	(− 0.12 to − 0.02)	–	–
AGE^2^	0.03	(0.02 to 0.04)	0.008	(0.003 to 0.013)	0.01	(0.007 to 0.014)	0.01	(0.01 to 0.02)	–	–

Extremely preterm participants only					
SDQ Total Difficulties Score adjusted for CBCL at 2.5 years (*n* = 235)			SDQ Total Difficulties Score adjusted for moderate/severe cognitive impairment at last clinical assessment (*n* = 249)		
Parameter	Estimate	95% CI	Parameter	Estimate	95% CI
Constant	10.84	(8.99 to 11.81)	Constant	10.37	(9.22 to 11.51)
AGE	− 0.37	(− 0.60 to − 0.14)	Age	− 0.33	(− 0.55 to − 0.10)
AGE^2^	0.03	(0.01 to 0.04)	Age^2^	0.02	(0.01 to 0.04)
CBCL clinically significant at 2.5 years	6.90	(5.10 to 8.70)	Moderate/severe cognitive impairment at last assessment	4.27	(2.76 to 5.77)

EPT is the group term that tests for the difference in scores at age 6 between the EPT and control group (intercept)

The AGE term is the increase in scores per year of age from the age of 6

The EPT*AGE term tests whether the scores in EPT and control group have different trajectories over time (slope)

The AGE^2^ term tests whether the trajectories depart from a straight linear line (curvature)

For example, for the SDQ total difficulties score: The EPT group score is 4.81 points higher than the control group mean score of 7.62 at age 6 and scores decrease in both groups by around 0.4 points per year of age; however, there is no difference in the slope of the trajectories between groups as the EPT*AGE term is not significant

*CBCL* child behavior checklist, *EPT* extremely preterm, *SDQ* strengths and difficulties questionnaire

### SDQ subscale scores in extremely preterm individuals and term-born controls

The subscale scores and impact score with 95% confidence intervals at each age are presented in Table 1 and displayed in Fig. 1b–f. The estimated coefficients and 95% confidence intervals are presented in Table 2. There was a significant difference between the EPT and control group for every subscale at age 6 (all *p* ≤ 0.001) (Table 2), although the difference seen in the Total Difficulties score was mainly driven by hyperactivity/inattention (Mean difference: 2.33, 95% CI 1.84–2.83) and peer relationship problems (Mean difference: 1.37, 95% CI 1.05–1.68). Both groups displayed a similar trajectory for peer relationship problems but there was evidence of a slight decline in hyperactivity/inattention problems in the EPT group over time. There was evidence of a different trajectory of emotional symptoms between groups which, although increased in both groups with age, this was to a greater degree in the EPT individuals. The mean score for conduct problems was marginally elevated in the EPT group and showed a decline relative to the controls over time. The EPT group was at increased risk of being classified as clinically significant for all domains (Fig. 2b–e), and the risk of having behavioral problems that have a substantial impact on home life, friendships, school or work and/or leisure activities was also significantly greater for EPT participants compared to term-born controls (Risk ratio: 4.28, 95% CI 2.89–6.35, *p* < 0.001) (Fig. 2f).

### SDQ Total Difficulties Score adjusted by CBCL clinical classification in infancy and moderate/severe cognitive development at last assessment

The SDQ Total Difficulties Scores in EPT participants stratified by Child Behavior Checklist classification at age 2.5 and moderate/severe cognitive impairment at last assessment are shown in Table 3. A CBCL classification of clinical behavioral problems at age 2.5 years was strongly associated with a high SDQ Total Difficulties score from 6 years onwards, with average scores 6.9 points higher compared to participants with a normal/borderline classification (95% CI 5.01–8.70, *p* < 0.0.01) (Table 2, Fig. 1g) and trajectories were similar in the normal/borderline and clinical group. Moderate/severe cognitive impairment at the last clinical assessment was also associated with a higher Total Difficulties score in EPT participants (Mean difference 4.27, 95% CI 2.76–5.77, *p* < 0.001); however, it did not account for all of the difference in scores compared to term-born peers. The mean Total Difficulties score in EPT children with mild or no cognitive impairment was still elevated at 10.37 (95% CI 9.22–11.51) compared to 7.62 (95% CI 6.87–8.38) in the term-born control group (Table 2, Fig. 1h). Both CBCL classification and cognitive impairment remained significant when added to the same model (both *p* < 0.001).

**Table 3:** SDQ total difficulties scores in extremely preterm participants by age of assessment, stratified by child behavior checklist classification at age 2.5 and moderate/severe cognitive impairment at last assessment

	Age 6 years (*n* = 241)	Age 11 years (*n* = 219)	Age 16 years (*n* = 138)	Age 19 years (*n* = 127)
CBCL at age 2.5 years				
Normal/borderline				
No. (%)	167 (79.1)	160 (79.6)	107 (80.5)	93 (82.3)
Mean (95% CI)	10.7 (9.8–11.6)	9.3 (8.3–10.3)	10.6 (9.3–11.9)	11.1 (9.7–12.4)
Clinical				
No. (%)	44 (20.9)	41 (20.4)	26 (19.5)	20 (17.7)
Mean (95% CI)	17.9 (16.0–19.9)	17.0 (14.6–19.4)	17.4 (14.1–20.8)	17.0 (14.0–20.0)
Mean difference (95% CI)	7.2 (5.2–9.2)	7.7 (5.4–10.0)	6.8 (3.8–9.9)	5.9 (2.7–9.1)
Cognitive impairment				
No/mild impairment				
No. (%)	117 (53.2)	118 (56.5)	76 (56.7)	63 (54.3)
Mean (95% CI)	10.9 (9.7–12.1)	9.0 (7.8–10.3)	9.7 (8.3–11.2)	10.4 (8.7–12.1)
Moderate/severe impairment				
No. (%)	103 (46.8)	91 (43.5)	58 (43.3)	53 (45.7)
Mean (95% CI)	13.8 (12.5–15.0)	13.8 (12.3–15.3)	14.7 (12.7–16.8)	14.3 (12.5–16.1)
Mean difference (95% CI)	2.9 (1.1–4.6)	4.8 (2.8–6.8)	5.0 (2.6–7.5)	3.9 (1.4–6.4)

Higher scores indicate more behavioral problems

*CBCL* child behavior checklist, *EPT* extremely preterm, *SDQ* strengths and difficulties questionnaire

### Complete-case analysis

All analyses were repeated for the 119 completers (82 EPT and 37 controls) and the results are shown in Online Resources Table S3 and Figure S2. The SDQ Total Difficulties Score and the four component subscales were slightly lower (indicating fewer behavioral problems) in the complete-case analysis compared to the analysis including all participants, but otherwise, the overall results were consistent with the primary analysis.

## Discussion

This study is the first to investigate trajectories of behavior, attention, social and emotional problems from childhood to young adulthood in EPT survivors and their term-born counterparts. Consistent with previous cross-sectional reports [2, 12, 14, 30], the results show that, overall, EPT individuals have significantly more behavioral problems than term-born peers at 6 years of age and this difference remains stable throughout adolescence and into young adulthood. In addition, we have shown that, although on a different level, the trajectories of overall behavioral problems are similar in both EPT and term-born individuals. However, the assessment of overall behavioral problems can mask between-group differences in separable symptom domains, as is demonstrated in the present study. The higher prevalence of overall problems was driven by the significant excess of emotional symptoms, hyperactivity/inattention and peer relationship problems in the EPT group, which is consistent with the triad of problems that characterizes the preterm behavioral phenotype [6]. Moreover, we observed differences in trajectories both between symptom domains and between groups.

For hyperactivity/inattention, scores were persistently higher among the EPT individuals but symptoms decreased slightly over time. The decline in ADHD symptoms and disorders with age for both term born and individuals with very low birth weight has been noted in previous studies which have also shown that the between-group difference in ADHD symptoms declines over time [2, 30]. We have shown that the group difference in ADHD symptoms (SDQ scores) remains similar over time but the risk for clinically significant problems in EPT individuals declines from childhood to young adulthood.

For peer relationship problems, trajectories in SDQ scores were similar between groups with persistent between-group differences across childhood and adolescence, but an increased risk for clinically significant problems among EPT individuals over time compared with those born at term, which peaked at age 16. Peer problems, in particular avoidant personality have been reported into early adulthood [31, 32]. For emotional problems, trajectories were different with a greater increase in SDQ scores among the EPT children and an increased risk for clinically significant problems over time. This may reflect the generally later onset of emotional problems in adolescence observed in the natural course of these disorders. It appears that EPT individuals may be at increased risk of emotional problems still in adulthood compared to studies who reported on VPT young adults [32].

The mechanisms underlying the neurobehavioral profile of EPT survivors are still unclear, although several explanations have been proposed [33, 34]. Neurodevelopmental immaturity at birth and altered brain development superimposed with neonatal brain injuries might be the main contributors postnatally, mediated by cognitive deficits and environmental influences. In studies of children and adolescents born preterm, the increased risk of internalizing behaviors and attention deficits have been linked to structural brain abnormalities [35]. The stressful environment of a busy neonatal unit and exposure to frequent and often painful therapeutic interventions may disrupt normal neurodevelopment, even in the absence of focal brain injury. Reduced opportunity for parent–infant interaction and limited vocal/visual stimulation may explain the observed problems in social interaction after discharge [34]. Later environmental influences in early infancy and childhood, such as parental mental health, caregiving style, or limited contact with peers and family due to prolonged periods of hospitalization/illness, may impede the development of coping strategies, emotional regulation, attachment, and other social skills in children born EPT [7, 36]. Core deficits in working memory have been implicated in the inattention difficulties observed in children born EPT [37, 38]. Also, hyperactive behaviors have been shown to be mediated by general intellectual delay, and sensitive/isolated behaviors (as evaluated by peers) associated with neuromotor delay [37].

Generally, the greater persistence of problems over time in this study compared with other very and extremely low birth weight cohorts [2, 14, 16, 30, 39] may also be related to the greater neurodevelopmental immaturity at birth, higher risk for neonatal brain injury and reduced neurodevelopmental plasticity that is conferred by birth at extremely low gestations compared with birth after 26 weeks of gestation. Indeed, moderate/severe cognitive impairment, an index of such factors [40, 41], accounted for some of the difference in the overall behavioral problems with those having cognitive difficulties having persistently greater problems, but did not explain the excess entirely. There is growing interest in the developmental mechanisms for these disorders and evidence that cognitive deficits such as executive function and processing speed, for example, might mediate the relationship between preterm birth and behavioral, social and emotional outcomes in early and mid-childhood [42, 43]. Early cognitive interventions may, therefore, impact on later behavioral, social and emotional outcomes and may be a fruitful avenue for future research.

We also found that EPT participants were over four times more likely to have behavioral problems that had a significant detrimental impact on their home life, friendships, and school and leisure activities compared with term-born controls. Attenuating the risk of behavioral problems and improving social skills by providing intervention for those with symptoms should, therefore, be a key aim of clinical follow-up. A clinical classification of behavioral problems at 2.5 years on the CBCL was found to be strongly associated with an increased risk of behavioral problems in later childhood. An association between parent-reported early childhood behavior or emotional problems and later psychiatric problems and disorders has also been shown in this and other EPT cohorts [44–46]. Given the stability of behavioral problems from childhood to young adulthood shown here, and the increasing risk for emotional problems over time, screening for behavioral and emotional problems in early childhood would be a valuable strategy for identifying those at risk and for facilitating the provision of early intervention or support.

### Strengths and limitations

A major strength of this large dual nation study of EPT survivors is its population-based design; findings are based on a prospective cohort of all live births in all centers in a geographically defined region and participants were followed up longitudinally from age 6 to age 19. In addition, longitudinal statistical analysis techniques were employed to examine behavioral trajectories of from early childhood to adulthood, which has many advantages over cross-sectional analysis techniques that simply compare group averages at each time point. In common with other longitudinal studies, the number of participants lost to follow-up increased over time, and was related to markers of social disadvantage and disability, though there was no evidence of a difference in CBCL scores at age 2.5 between participants with complete and incomplete data. Our findings were strengthened by the analysis of individuals with complete follow-up which corroborated the main results, although this subset of participants had a lesser degree of behavioral difficulties in general compared to all participants.

## Conclusion

Attention and peer relationship problems in extremely preterm individuals persist into early adulthood, and emotional symptoms increase as they enter adolescence. The increased risk of clinically significant behavioral problems continues to have a substantial impact on their everyday lives as young adults. A positive behavioral screen in infancy and moderate/severe cognitive impairment are strongly associated with early adult outcomes, and the introduction of screening into routine clinical follow-up may enable early interventions for those most likely to benefit.

## Electronic supplementary material

Below is the link to the electronic supplementary material.

Supplementary material 1 (DOCX 114 kb)
