# Supplementary material for: Trajectories of behavior, attention, social and emotional problems from childhood to early adulthood following extremely preterm birth: a prospective cohort study
Source: Eur Child Adolesc Psychiatry. 2018 Sep 7;28(4):531–42. doi: 10.1007/s00787-018-1219-8 (PMC6445809; doi:10.1007/s00787-018-1219-8)
Supplement: Supplementary file 1 — Supplementary material 1 (DOCX 114 kb) [file 787_2018_1219_MOESM1_ESM.docx]

**Supplementary Material**

**Trajectories of behavioural problems from childhood to early adulthood following extremely preterm birth: a prospective cohort study**

**Authors:**

Louise Linsell^1^, BSc MSc PhD

Samantha Johnson^2^, BSc PhD

Dieter Wolke^3^, PhD Dr Rer Nat H C

Joan Morris^4^, MA MSc PhD

Jennifer J Kurinczuk^1^, BSc MBChB MSc MD FFPH

Neil Marlow^5^, BA MBBS MRCP MD FRCPCH FMedSci

**Affiliations:**

^1^ National Perinatal Epidemiology Unit (NPEU), Nuffield Department of Population Health, University of Oxford, Old Road Campus, Headington, Oxford, OX3 7LF.

^2^ Department of Health Sciences, University of Leicester, Centre for medicine, University Road, Leicester LE1 7RH.

^3^ Department of Psychology and Division of Health Sciences, Warwick Medical School, University of Warwick, Coventry, CV4 7AL.

^4^ Queen Mary University of London, Centre for Environmental and Preventive Medicine, Barts and The London School of Medicine and Dentistry, Charterhouse Square, London, EC1M 6BQ.

^5^ Institute of Women's Health, University College London, Medical School Building, 74 Huntley Street, London, WC1E 6AU.

**Address correspondence and reprint requests to:** Dr Louise Linsell, National Perinatal Epidemiology Unit (NPEU), Nuffield Department of Population Health, University of Oxford, Old Road Campus, Headington, Oxford, OX3 7LF [louise.linsell@npeu.ox.ac.uk].

**Contents**

[Figure S1: Flow of study participants in the EPICure cohort study 3](#_Toc518645963)

[Table S1: Perinatal and neurodevelopmental characteristics of extremely preterm participants according to completeness of behavioural screening assessments 4](#_Toc518645964)

[Table S2: Characteristics of term-born controls according to completeness of behavioural screening assessments 5](#_Toc518645965)

[Table S3: Complete case: mixed model analysis of the SDQ Total Difficulties Score and subscales in extremely preterm participants and term-born controls 6](#_Toc518645966)

[Figure S2: Complete case: mean plus 95% confidence intervals for SDQ Total Difficulties and subscale scores in EPT participants and term-born controls at age 6, 11, 16 and 19 7](#_Toc518645967)

# Figure S1: Flow of study participants in the EPICure cohort study

| **Extremely preterm participants Term-born controls**  812 infants <26 completed weeks' gestation born between 1st March to 31st December 1995  **Delivery**  **Discharge**  315 alive at hospital discharge  6 died  **2.5 years**  283/309 (92%) assessed  **6 years**  160 matched controls  2 died  241/309 (78%) assessed  153 matched controls  110 previously assessed  43 new controls  **11 years**  219/307 (71%) assessed  **16 years**  138/307 (45%) responded to postal questionnaire  88 matched controls  65 matched controls  129/306 (42%) assessed  1 died  **19 years** |
| --- |

# Table S1: Perinatal and neurodevelopmental characteristics of extremely preterm participants according to completeness of behavioural screening assessments

|  | **Completers:**  **4 assessments**  **(n=82)** | **Non-completers**  **<4 assessments**  **(n=233)** | | **P value^a^** |
| --- | --- | --- | --- | --- |
| **Maternal/paternal factors**  Maternal age (years), mean [SD]: | 29.4 [5.6] (n=81) | 28.1 [6.0] (n=232) | | 0.10 |
| Mother of non-white ethnicity, % (n): | 12.4% (10/81) | 27.0% (63/233) | | 0.01 |
| Primigravida, % (n): | 37.8% (31/82) | 27.6% (64/232) | | 0.09 |
| Mother smoked during pregnancy, % (n): | 24.7% (20/81) | 36.5% (73/200) | | 0.07 |
| Antenatal steroids given, % (n): | 81.7% (67/82) | 77.9% (180/231) | | 0.53 |
| Mother's highest educational qualification A ‘level  or above, % (n):^b^ | 47.5% (38/80) | | 31.4% (61/194) | 0.01 |
| Father's occupation non-manual, % (n):^b^ | 54.2% (39/72) | | 29.1% (46/158) | <0.001 |
| **Infant perinatal factors**  Multiple birth, % (n): | 32.9% (27/82) | | 22.4% (52/232) | 0.08 |
| Congenital anomaly present, % (n): | 2.4% (2/82) | | 2.6% (6/232) | 1.00 |
| Gestational age 24 weeks or less, % (n): | 46.3% (38/82) | | 39.1% (91/233) | 0.30 |
| Birthweight (grams), mean [SD]: | 733 [120] (n=82) | | 752 [112] (n=233) | 0.19 |
| Male sex, % (n): | 41.5% (34/82) | | 51.9% (121/233) | 0.12 |
| Moderate/Severe brain injury during neonatal period, % (n):^c^ | 17.1% (14/82) | | 24.6% (57/232) | 0.22 |
| Laser or cryotherapy for retinopathy of prematurity, % (n): | 13.4% (11/82) | | 14.9% (34/228) | 0.86 |
| Laparotomy for necrotising enterocolitis, % (n): | 2.5% (2/79) | | 3.1% (7/229) | 1.00 |
| Supplemental oxygen at 36 weeks, % (n): | 76.8% (63/82) | | 73.0% (170/233) | 0.56 |
| **Infant developmental outcomes at 2.5 years** |  | |  |  |
| BSID-II Mental Developmental Index, mean [SD]: | 84.5 [12.7] (n=82) | | 76.5 [17.3] (n=201) | <0.001 |
| BSID-II Psychomotor Developmental Index, , mean [SD]: | 86.3 [15.1] (n=80) | | 78.7 [19.0] (n=182) | 0.002 |
| BSID-II Behaviour Rating Percentile, , mean [SD]: | 39.2 [26.1] (n=79) | | 34.6 [24.9] (n=180) | 0.18 |
| CBCL Total Problem T-score, mean [SD]: | 54.5 [9.2] (n=82) | | 56.2 [9.9] (n=188) | 0.17 |
| **Neurodevelopmental impairment at last assessment^b^**  Cerebral palsy, % (n): | 7.3% (6/82) | | 22.2% (50/225) | 0.002 |
| Moderate/severe cerebral palsy, % (n): | 4.9% (4/82) | | 14.0% (31/222) | 0.03 |
| Moderate/severe cognitive impairment, % (n): | 45.1% (37/82) | | 49.1% (103/210) | 0.60 |
| Moderate/severe visual impairment, % (n): | 7.3% (6/82) | | 12.1% (27/224) | 0.30 |
| Moderate/severe hearing impairment, % (n): | 1.2% (1/82) | | 2.2% (5/225) | 1.00 |
| Moderate/severe functional impairment, % (n):^d^ | 46.3% (38/82) | | 53.3% (112/210) | 0.30 |
| Severe functional impairment, % (n):^d^ | 18.3% (15/82) | | 27.1% (57/210) | 0.13 |
| BSID-II Bayley Scales of Infant Development (second edition); CBCL Child Behaviour Checklist; EPT Extremely preterm.  ^a^ Two-sided p-values were calculated using Fisher's Exact Test for binomial variables and the t-test for continuous variables.  ^b^ Not collected at discharge; first collected at one or two year assessment.  ^c^ Parenchymal pathology and/or ventriculomegaly on worst cranial ultrasound scan before discharge home.  ^d^ Functional impairment includes cerebral palsy, cognitive, visual or hearing impairment at clinical assessment. | | | | |

# Table S2: Characteristics of term-born controls according to completeness of behavioural screening assessments

|  | **Completers:**  **4 assessments**  **(n=37)** | **Non-completers**  **<4 assessments**  **(n=169)** | **P value^a^** |
| --- | --- | --- | --- |
| Mother's highest educational qualification A ‘level or above, % (n): | 50.0% (18/36) | 38.5% (57/148) | 0.26 |
| Male sex, % (n): | 40.5% (15/37) | 43.2% (73/169) | 0.86 |
| Any cognitive impairment at last assessment, % (n) | 8.1% (3/37) | 20.5% (34/166) | 0.10 |
| Moderate/severe cognitive impairment at last assessment, % (n): | 0.0% (0/37) | 3.0% (5/166) | 0.59 |
| Any visual impairment at last assessment, % (n): | 32.4% (12/37) | 18.2% (30/165) | 0.07 |
| Any hearing impairment at last assessment, % (n): | 2.7% (1/37) | 0.0% (0/165) | 0.18 |
| Any functional impairment at last assessment, % (n):^b^ | 40.5% (15/37) | 34.6% (57/165) | 0.57 |
| ^a^ Two-sided p-values were calculated using Fisher's Exact Test.  ^b^ Functional impairment includes cerebral palsy, cognitive, visual or hearing impairment at clinical assessment. | | | |

# Table S3: Complete case: mixed model analysis of the SDQ Total Difficulties Score and subscales in extremely preterm participants and term-born controls

| **Extremely preterm participants compared to term-born controls** | | | | | | | | | | | | | | |
| --- | --- | --- | --- | --- | --- | --- | --- | --- | --- | --- | --- | --- | --- | --- |
| **Parameter** | **SDQ Total Difficulties Score (n=119)** | | | **Emotional Symptoms Score (n=119)** | | | **Conduct Problems Score (n=119)** | | | **Hyperactivity/Inattention Score (n=119)** | | | **Peer Problems Score**  **(n=119)** | |
|  | **Estimate** | **95% CI** | | **Estimate** | | **95% CI** | **Estimate** | | **95% CI** | **Estimate** | **95% CI** | | **Estimate** | **95% CI** |
| Constant  EPT  Age  Age^2^ | 6.11  6.21  -0.45  0.03 | (4.69 to 7.53)  (4.32 to 8.10)  (-0.69 to -0.20)  (0.02 to 0.05) | | 1.49  1.39  -0.10  0.01 | | (0.97 to 2.02)  (0.77 to 2.00)  (-0.21 to 0.003)  (0.004 to 0.02) | 1.58  0.30  -0.19  0.01 | | (1.10 to 2.06)  (-0.21 to 0.80)  (-0.26 to -0.11)  (0.007 to 0.017) | 2.64  2.54  -0.21  0.01 | (1.92 to 3.36)  (1.74 to 3.35)  (-0.32 to -0.11)  (0.003 to 0.02) | | 0.57  1.75  0.05  - | (0.20 to 0.94)  (1.18 to 2.32)  (0.02 to 0.08)  - |
| **Extremely preterm participants only** | | | | | | | | | | | | | | |
| **SDQ Total Difficulties Score adjusted for CBCL at 2.5 years (n=82)** | | | | | | | | **SDQ Total Difficulties Score in adjusted for moderate/severe cognitive impairment at last clinical assessment (n=82)** | | | | | | |
| **Parameter** | | | **Estimate** | | **95% CI** | | | **Parameter** | | | **Estimate** | **95% CI** | | |
| Constant  Age  CBCL clinically significant at 2.5 years | | | 10.40  -0.007  8.53 | | (9.90 to 11.77)  (-0.10 to 0.09)  (5.17 to 11.89) | | | Constant  Age  Moderate/severe cognitive impairment at last assessment | | | 10.22  -0.007  3.39 | (8.31 to 12.12)  (-0.10 to 0.09)  (0.69 to 6.10) | | |
| CBCL Child Behaviour checklist; EPT Extremely preterm; SDQ Strengths and Difficulties Questionnaire. | | | | | | | | | |  | | |  | |

# Figure S2: Complete case: mean plus 95% confidence intervals for SDQ Total Difficulties and subscale scores in EPT participants and term-born controls at age 6, 11, 16 and 19

| Total Difficulties Score   | Emotional Symptoms Score   |
| --- | --- |
| Conduct Problems Score   | Hyperactivity/Inattention Score   |
| Peer Problems Score   | Impact Score   |
|  |  |
| g) Total Difficulties Score in EPT participants stratified by CBCL score at 2.5 years   | h) Total Difficulties Score in EPT participants stratified by moderate/severe cognitive impairment   |
